# Intraoperative neurophysiologic monitoring alteration during en bloc laminectomy surgery for thoracic ossification of ligamentum flavum

**DOI:** 10.3389/fsurg.2022.1019112

**Published:** 2022-09-27

**Authors:** Xiaoning Feng, Li Deng, Haoyu Feng, Yong Hu, Jianghua Tian, Lin Sun

**Affiliations:** ^1^ Department of Orthopedics, Shanxi Bethune Hospital, Shanxi Academy of Medical Sciences, Tongji Shanxi Hospital, Third Hospital of Shanxi Medical University, Taiyuan, China; ^2^ Department of Orthopaedics and Traumatology, The University of Hong Kong, Pokfulam, Hong Kong SAR, China; ^3^ Department of Orthopedics, Second Hospital of Shanxi Medical University, Taiyuan, China

**Keywords:** intraoperative neurophysiologic monitoring, thoracic ossification of ligamentum flavum, motor-evoked potential, somatosensory-evoked potential, spinal decompression, prognosis

## Abstract

**Background:**

There is real risk during *en bloc* resection for the treatment of thoracic ossification of ligamentum flavum (TOLF). Intraoperative neurophysiologic monitoring (IONM) has been applied to monitor neurologic functional integration of the spinal cord during surgery. However, the IONM outcome and its relationship with clinical results still needs to be investigated. The purpose of this study is to evaluate the effectiveness and usefulness of IONM in *en bloc* laminectomy for TOLF.

**Methods:**

Data from a total of 68 patients with TOLF who received *en bloc* resection was collected for this retrospective study. IONM of somatosensory-evoked potentials (SSEPs) and motor-evoked potentials (MEPs) were analyzed in different patterns of signal alerts, i.e. alert in either MEPs or SSEPs, alert in both MEPs and SSEPs, permanent alert, or recovery during surgery. Postoperative motor and sensory neurological function was evaluated in each patient immediately after surgery and at 12-month follow-up after surgery. The relationship of IONM outcomes and postoperative neurologic function were observed.

**Results:**

Fifty of 68 patients did not present significant changes over alert criteria of IONM, neither SSEPs nor MEPs. Those 50 patients without IONM alerts did not show post-operative neurologic deterioration. Four patients presented alert of IONM in a single modality, while 2 patients showed only SSEP alert and 2 patients showed only MEP alert. Fourteen patients showed alerts in both SSEP and MEP, while 8 patients showed one or both signals return to normal during surgery and other 6 showed permanent abnormal electrophysiologic signals to the end of surgery. In the follow-up neurologic test, 3 patients presented transient neurologic complications from among 8 patients with both SSEP and MEP alerts and recovery during surgery. Six patients without recovery of IONM showed permanent neurologic complications after surgery.

**Conclusions:**

Results of this study prove the effectiveness and usefulness of IONM in in *en bloc* laminectomy surgery for TOLF. The patterns of IONM changes correlated with postoperative neurologic functions. Special attention must be paid to the rapid loss of IONM without recovery during spinal decompression.

## Introduction

The most common pathologic type of thoracic spinal stenosis (TSS) is thoracic ossification of ligamentum flavum (TOLF) (1–3). Ossification of the ligamentum flavum (OLF) has been reported in more than 50% of surgical cases for degenerative TSS (4). Because OLF is a symptom that may progress as the ossification expands, early decompressive surgery is a better option than continued conservative treatment for no purpose. The most common procedure is posterior resection of the ossified area and affected lamina. Surgical approaches include *en bloc* laminectomy, open-door laminectomy, hemilaminectomy, and fenestration (4, 5). In addition to postoperative recovery, clinical and cost effectiveness are also important in the choice of surgical approach (6). Under comprehensive evaluation, the *en bloc* laminectomy approach is the most effective and appropriate thoracic decompression surgery to treat TOLF (7). However, the *en bloc* laminectomy is usually accompanied by the risk of postoperative neurologic deterioration (8). Intraoperative neurophysiologic monitoring (IONM) is recommended to facilitate timely recognition of intraoperative spinal cord injury (9).

IONM of both motor-evoked potentials (MEPs) and somatosensory-evoked potentials (SSEPs) is an effective and accurate monitoring method for determining nerve injury during spinal surgery (10, 11). Although there are currently no specialized studies of IONM in *en bloc* laminectomy resection of TOLF, increasing evidence supports its use in spinal surgery (12, 13). However, the absence of an effective intraoperative alert management protocol in surgical treatment indicates a key technical deficiency. As research progresses, intraoperative management in the future will focus on developing treatments, validating practice protocols, and synthesizing clinical guidelines.

IONM has been widely utilized to detect injuries or damage to the spinal cord during posterior decompressive surgery (14–16) and in the treatment of degenerative cervical and thoracic spondylosis. Improved multimodal IONM in posterior cervical surgery achieved 100% sensitivity and 98.4% specificity in predicting postoperative defects, helping to reduce the incidence of postoperative neurologic deficits (17). According to previous research, the precise prognosis for postoperative neurologic status may depend on the different changes in IONM features and type of surgery performed (18, 19). However, TOLF is a relatively uncommon disease, and there are no reports on the use of IONM during *en bloc* laminectomy resection for TOLF. The purpose of this study was to determine the correlation of postoperative neurologic recovery in the management of TOLF with *en bloc* laminectomy and use of IONM.

## Patients and methods

### Patients

A total of 68 patients with TOLF that received *en bloc* removal surgery in the Spine Research Center from September 2012 to November 2021 were recruited for this study. All the surgeries were under intraoperative neuromonitoring of both SSEPs and MEPs. The inclusion criteria were (1) clinical signs of at least one symptomatic myelopathy; (2) evident signs of spinal cord compression on computed tomography or magnetic resonance imaging; (3) no medical history of surgical treatment of myelopathy; (4) absence of lumbar or cervical spinal stenosis. The exclusion criteria were (1) history of spinal disease or spinal surgery; (2) inoperability due to underlying disease; (3) patients with spinal trauma; (4) cases lost to follow-up. This study was approved by the Medical Ethics Committee with the ethics batch number: YXLL-2022-067.

### Surgical approach

All surgeries were performed in the same way. All patients were placed in the prone position, and a median incision was performed after general anesthesia. Target laminae and single-level lamina located both caudally and cephalad adjacent to the target segment were exposed to the junction of articular process and lamina. Resections of supraspinous and interspinous ligaments were carried out at both the inferior margin of the caudal lamina and superior margin of the cephalic lamina. Bilateral gutters into the dural sac at the laminae of target segments were made with a high-speed drill. Elevation of the spinous processes and laminae of target segments, and *en bloc* removal of excised laminae, spinous processes, and OLF were performed. Twenty-six patients underwent posterior fixation and fusion at the same time, due to multisegment laminectomy (more than two segments) (*N* = 7) or hypermobility after decompression at the junction of cervicothoracic and thoracolumbar spine (*N* = 19).

### Anesthesia management

When performing the IONM test, the anesthesia protocol was tightly controlled. The patient received an IONM-compatible intravenous anesthetic (propofol 5 g/ml and/or remifentanil 1.7 ng/ml) and a short-acting muscle relaxant (rocuronium 1 mg/kg) during intubation, but they were not used during surgery. After completion of tracheal intubation, neostigmine 2 mg was administered by an anesthesiologist to reverse the muscle relaxant effect.

### Intraoperative neurophysiologic monitoring

MEP and SSEP monitoring during posterior spinal decompression surgery has been well documented previously (16, 20). Before total laminar decompression, MEPs and SSEPs collected using hypodermic needle electrodes and patch electrodes were used as baseline data. The MEP parameters were as follows: 2.5–4.0 ms stimulus interval, 5–7 pulses, 200–400 µs duration, 150–300 V constant voltage, as well as placement of stimulating electrodes in the C3-C4 cortical region. The following muscles were selectively used for recording MEPs: deltoid, biceps, triceps, abductor pollicis brevis, quadriceps femoris, biceps femoris, tibialis anterior, gastrocnemius, abductor pollicis, and sphincter. The SSEP parameters were as follows: 200–300 µs duration, 5.0 and 5.6 Hz frequency, 25–35 mA constant current over the tibial/median nerve, single pulse, Cz and Fpz for lower extremity cortical SSEP recordings, C3 and C4 for upper extremity cortical SSEP recordings, 30–300 Hz bandpass filter, 50 ms window for upper extremities, 100 ms window for lower extremities, and average after 300 stimulations.

In our study, both general and anesthesia-induced changes in MEPs were first excluded through a unified anesthesia protocol, and MEP warning criteria were associated with a rapid loss of more than 80% of MEP amplitudes associated with nerve decompression (21). We set two mean baseline values where a 50% reduction in primary SSEP amplitude or the SSEP incubation period was significantly prolonged by more than 10% (22, 23).

### Clinical outcomes

The patterns of IONM were analyzed in two aspects. The first aspect was the consideration of monitoring modality, such that the IONM alerts were observed in either SSEPs or MEPs, or both SSEPs and MEPs. The second aspect was to consider the change pattern, if the IONM alerts recovered during surgery. The neurologic status was assessed in all patients at three time points: preoperatively, immediately after surgery, and at 12 months follow-up.

### Neurologic and clinical outcome assessment

The thoracic spinal cord function score of the Japanese Orthopaedic Association (JOA) (maximum score 11) was used to evaluate neural functions for patients with thoracic myelopathy (24).

### Statistical analysis

SPSS 26.0 statistical software (IBM Corp., Armonk, NY, USA) was used for data processing. All quantitative data are expressed as mean and standard deviation (SD), and a *P* value of <0.05 was considered statistically significant. One-way analysis of variance was used to compare JOA score between the no significant changes of IONM (N) group, IONM alarm in single modality (SM) group, IONM alarm in dual modality and then IONM alert recovery (DMR) group, and IONM alarm in dual modality and then IONM alert no recovery (DMNR) group before operation, after operation, and at last follow-up (12 months).

## Results

### Participants and descriptive data

The clinical characteristics of patients with TOLF are shown in Table 1. Patients with OLF of the thoracic spine were mainly distributed in the middle-aged and elderly population, and the average operation time was about 230 min. The duration of symptoms and the amount of intraoperative blood loss varied greatly among different patients. We found that patients with more intraoperative blood loss had higher rates of IONM alarms, and there may be a correlation between intraoperative MEP or SEP alarms and intraoperative blood loss.

**Table 1:** Basic characteristics of patients with thoracic ossification of the ligamentum flavum.

Characteristic	Mean ± SD	Range (min–max)
Age, years	55.5 ± 7.0	39–69
Height, cm	165.6 ± 8.6	145–181
Weight, kg	62.3 ± 11.6	40–91
BMI, kg/m^2^	22.6 ± 2.9	16.7–30
Operation time, min	230 ± 26.9	178–282
Duration of symptoms, months	25.4 ± 19.2	6–122
Bleeding volume, ml	648.5 ± 257.7	300–1400
Male: female	Male 39: female 27	

SD, standard deviation.

Values are presented as mean ± standard deviation (range), or number of cases.

### Treatment and grouping

**Figure 1:**
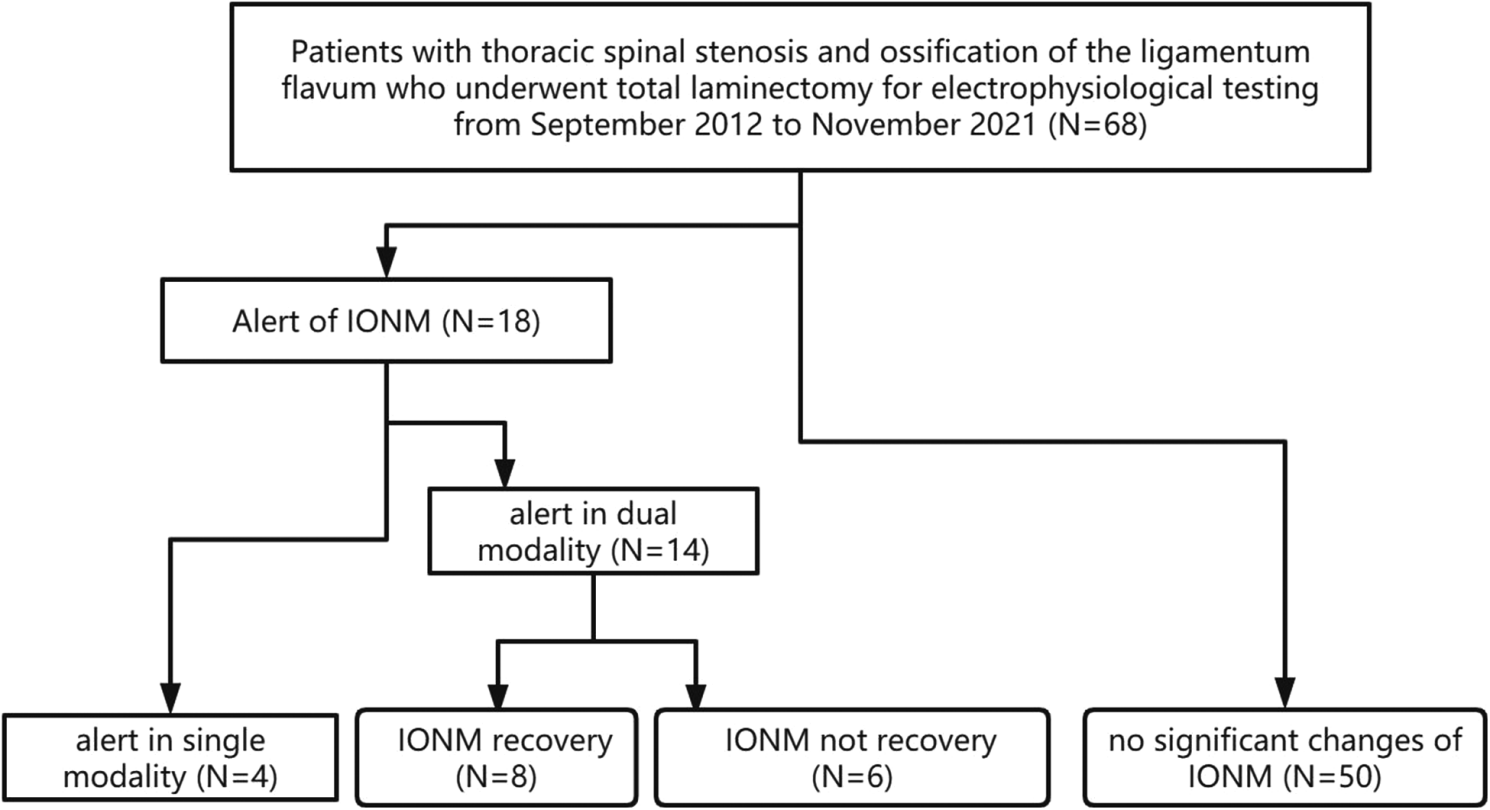
Flow diagram for patient inclusion. Alert in single modality: alert in SSEP or MEP IONM: intraoperative neurophysiologic monitoring.

A total of 68 TOLF patients undergoing *en bloc* laminectomy under IONM were collected between September 2012 to November 2021. Reliable IONM baselines (SSEPs or MEPs) were recorded in all 68 patients. A total of 50 patients showed no significant changes in SSEPs or MEPs. IONM alerts presented for 18 patients. Four patients presented alerts in single modality evoked potential, 2 had SSEP alerts, and the other 2 had MEP alerts. The other 14 patients had alerts of both MEPs and SEPs. Eight of 14 patients with alerts of both SSEPs and MEPs showed recovery of IONM after operative treatment, whereas the other 6 patients did not show recovery of IONM. The patients were divided into 4 groups (Figure 1) according to the IONM alarm status: SM, DMR, DMNR, and N (see section 2.7). For all patients with an IONM alarm, the operation was stopped immediately to avoid further stimulation of the spinal cord, exclude the interference of anesthesia factors, observe the partial pressure of oxygen by intraoperative blood gas analysis, allow the mean arterial pressure (MAP) to rise to 85 mmHg vasopressors, and give the patient methylprednisolone.

### De novo neurologic deficit

Neurologic function of every patient was evaluated immediately after surgery and at 12 months follow-up. In comparison to preoperative neurologic function, postoperative neurologic function assessment might identify new neurologic deficits. A transient neurologic complication was defined if the neurologic deficit was detected immediately after surgery but recovered within 12 months follow-up. Permanent neurologic injury was defined when the neurologic deficit was detected immediately after surgery to 12 months follow-up. In this study, 50 patients without IONM alerts did not experience postoperative neurologic deterioration. In contrast, their neurologic function was significantly recovered in comparison to their preoperative status. Four patients with either SSEP or MEP alerts did not show new neurologic complication after surgery. Five of 8 patients with alerts of both SSEP and MEP that resolved during surgery did not show new neurologic complications after surgery, whereas the other 3 patients showed transient neurologic complications. Six patients who had alerts of both SSEPs and MEPs without recovery of IONM presented permanent neurologic deficits after surgery (Table 2).

**Table 2:** New-onset neurologic deficit after surgery.

	N	SM	DMR	DMNR
Postoperative new nerve defect	0/50	0/50	3/8	6/6
New neurologic deficit persisted at last follow-up	0/50	0/50	0/8	6/6

### JOA score

**Figure 2:**
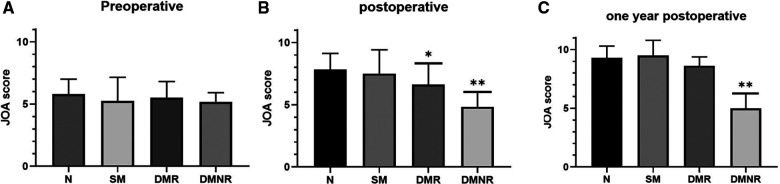
JOA score before and after surgery and at last follow-up. (**A**): JOA score of each group before surgery, no significant difference. *P < 0.05 and **P < 0.01; (**B**): The JOA score of the postoperative DMR group (*P* < 0.05) and DMNR group (*P* < 0.01) was lower than that of the normal group. (**C**): Compared with the N group, the JOA score decreased in the DMNR group one year after surgery (*P* < 0.01). JOA, Japanese Orthopaedic Association.

There was no statistically significant difference in the JOA scores between the 4 groups before surgery, indicating that the preoperative baseline characteristics of the 4 groups of patients were basically similar. The JOA score decreased in the DMR group (*P* < 0.05) and DMNR group (*P* < 0.01) after the operation (Figure 2). This indicates that compared with the normal group, alerts from dual modalities can indicate a relatively poor improvement in spinal cord function after surgery. One year after the operation, the JOA score of the DMNR group was lower than that of the normal group, indicating that intraoperative DMNR was associated with long-term spinal cord dysfunction.

### Typical case

**Figure 3:**
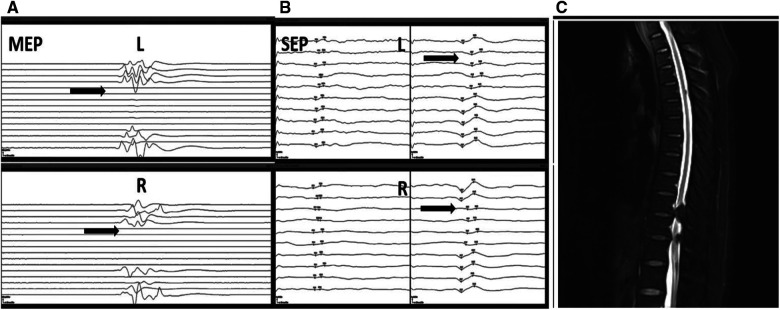
A typical sample case from a 59-year-old adult woman with T10-T12 TOLF with IONM alert and recovery. During the operation, the patient experienced rapid loss of MEP in both lower extremities (**A**). At the same time, the SSEP amplitude decreased by more than 50%, and the latency was prolonged by more than 10% (**B**). A dual-mode alarm signal appeared, and then IONM recovered. The patient's preoperative MRI images showed TOLF of the patient at T10-T12 with severe spinal cord compression (**C**). TOLF, thoracic ossification of ligamentum flavum; IONM, intraoperative neurophysiologic monitoring; MEP, motor-evoked potential; SSEP, somatosensory-evoked potential; MRI, magnetic resonance imaging.

A typical sample case (Figure 3) was a 59-year-old adult woman with T10-T12 TOLF and severe preoperative neurologic deficits. She underwent posterior thoracic spinal decompression (T10-T12), instrumentation (T9-L1), and posterolateral bone graft fusion. When decompressing the T10-T11 sections, bilateral MEP signal loss occurred immediately together with SSEP alerts. The surgeon immediately stopped the surgery to avoid further stimulation of the spinal cord, increased mean arterial pressure (MAP) to 85 mmHg, and administered methylprednisolone to the patient. After these series of measures, the IONM partially recovered and the surgery continued. After treatment, there was no new neurologic deficit in the patient from the operation to the last follow-up, and the JOA score increased significantly after the operation and at the last follow-up.

## Discussion

IONM for degenerative spinal decompression is a hotspot of current research and attention. Studies have shown that different levels of IONM alerts are important for the prognostic assessment of postoperative neurologic status (11). Patients with TOLF have a significantly higher risk of rapid loss of IONM and spinal complication compared with patients with other spinal degenerative diseases (25). The rapid loss of IONM without recovery during spinal decompression must be specifically addressed in the surgical management of myelopathy during TOLF surgery (26).

Our study reported that the occurrence rate of IONM alarms in TOLF surgery was 26.5%. In a comparison of intraoperative neuromonitoring results between *en bloc* and segmental laminectomy for OLF of the thoracic spine, the IONM alarm rate during *en bloc* laminectomy was 16.4% (7). We reported a higher incidence of intraoperative alarms in IONM, which may be related to the greater number of spinal cord decompression segments and greater intraoperative blood loss in the patients we included on average. According to our current study, IONM alerts during thoracic spinal decompression surgery are associated with a significantly increased risk of postoperative neurologic deficits. In this study, all patients with dual-mode alarm and non-recovery during operation developed new-onset permanent neurologic deficit, suggesting that dual-mode IONM alarm and non-recovery during thoracic spinal decompression surgery are closely related to permanent neurologic deficit. Therefore, spine surgeons must pay special attention to surgical procedures to reduce the iatrogenic risk of neurologic deficits during thoracic surgery. To take more precise measures toward avoiding injury aggravation, we can estimate the proportion of injuries to the spinal cord by the nerve conduction pathway. SSEPs directly assess the integrity of the dorsal part of the spinal cord, and MEPs assess the integrity of the anterior and lateral parts of the cord. Changes or alerts in the IONM waveform are very closely related to high-risk surgical procedures or interventions (11). Intraoperative changes of MEPs and SEPs potentially provide a valid method for quantitatively evaluating the safety of different intraoperative manipulations and their prognostic impacts on spinal cord (7). IONM alerts can detect nerve damage, indicate the need for prompt surgical intervention, and verify the prediction of postoperative neurophysiologic functional status based on IONM alerts.

Because patients with TOLF always have different levels of preoperative neurologic deficits, it is more difficult to acquire effective IONM signals than in cases with normal neurologic function, especially under general anesthesia. If poor IONM signals lead the surgical team to obtain false-positive IONM results, then the following incorrect surgical procedures or unnecessary wake-up tests will strongly interfere with the operation. Hence, stable and reliable IONM baseline values and improved accuracy are crucial, and effective treatment strategies help surgeons to choose the best surgical procedure to achieve the best treatment effect. To achieve this, qualified skills of the IONM team are needed in addition to propofol and remifentanil–based total intravenous anesthesia and appropriate depth of anesthesia. Hence, collaborative teamwork between anesthetists and neurophysiologists will be critical in TOLF surgery. In addition, the patients included in this study were all patients with degenerative thoracic vertebral disease, and patients with spinal trauma and other spinal diseases and history of previous spine surgery were excluded. The reason is that emergency scheduling of IONM in patients with spinal trauma can be difficult, and additional confounding factors can make the baseline unreliable (27).

Moreover, according to our experience, the rapid loss of IONM often reflects a severe neurological injury in TOLF. Previous studies also reported that the speed of intraoperative MEP reduction in the thoracic section was related to impending spinal hypofunction to a certain extent (26, 28). In addition, blood supply to the thoracic segment is worse than that to the cervical or lumbar segment; hence, the deterioration of IONM in the thoracic segment is more serious, which is also an important reason for the loss of IONM signal during thoracic decompression surgery (3). Thus, IONM reduction in the thoracic cord is probably rapid. To avoid missing IONM information among other parameters, we can take full advantage of the SSEP and MEP characteristics to identify neurologic deficits without delay. SSEPs are real-time continuous monitoring modes. At the same time, MEPs can be quickly and easily collected before and after critical surgery as needed. The combination of SSEPs and MEPs will better evaluate spinal cord function.

The development of spine surgery is inseparable from the development of a large number of technical assistance needs. The combined use of IONM and imaging monitoring can optimize the surgical approach, improve flexibility, and increase surgical safety.

Intraoperative ultrasound (IoUS) is currently the most commonly used method of intraoperative imaging monitoring due to its widespread availability and relative affordability (29). The application of IoUS can determine the scope of spinal cord decompression, exclude subdural hemorrhage, anterior spinal depression or spinal cord herniation, and can quickly find the cause of IONM alarm. Therefore, the combined application of various imaging and IONM in spinal surgery has good potential.

Sometimes, a complete loss of MEPs in the surgery of an intramedullary spinal cord tumor and in spinal osteotomy does not have to result in permanent paraplegia (30, 31). However, this opinion probably does not apply in patients with TOLF. TOLF mostly involves the presence of a preoperative neurologic deficit and easily leads to new deficit in the absence of a direct or indirect physical insult to the spinal cord during decompression surgery. Furthermore, because the thoracic spinal cord is much smaller in diameter than the cervical or lumbar spinal cord, there is much less blood supply at the thoracic level. Hence, an IONM reduction related to spinal decompression of a severely stenotic section should arouse our attention, which is probably predicted to relate to postoperative weakness or transient neurologic deficits.

In conclusion, our study reported the postoperative neurologic status of IONM alarms at different alarm levels. The different levels of IONM alerts have corresponding variation in prognosis for postoperative neurologic status in patients with TOLF. In patients who have no deterioration in either SEPs or MEPs, postoperative neurologic function is normal. In those with alerts in either MEPs or SEPs or both MEPs and SEPs, followed by recovery, neurologic function can be restored in some patients. In those who have alerts in both SEPs and MEPs without recovery, neurologic function must have been injured after surgery. More attention should be paid to patients with rapid loss of IONM without recovery during spinal decompression.

## Data Availability

The original contributions presented in the study are included in the article/Supplementary Material, further inquiries can be directed to the corresponding author/s.
